# Exploring fear of COVID-19 and its correlates among older adults in Bangladesh

**DOI:** 10.1186/s12992-021-00698-0

**Published:** 2021-04-14

**Authors:** Sabuj Kanti Mistry, A. R. M. Mehrab Ali, Farhana Akther, Uday Narayan Yadav, Mark F. Harris

**Affiliations:** 1 ARCED Foundation, 13/1, Pallabi, Mirpur-12, Dhaka, Bangladesh; 2 Centre for Primary Health Care and Equity, University of New South Wales, Sydney, Australia; 3 BRAC James P Grant School of Public Health, BRAC University, 68 Shahid Tajuddin Ahmed Sharani, Mohakhali, Dhaka, 1212 Bangladesh; 4 Innovations for Poverty Action, New Haven, USA; 5 Mawlana Bhashani Science and Technology University, Tangail, Bangladesh; 6 SPM department, Faculty of Medicine, University of Malaya, Kuala Lumpur, Malaysia

**Keywords:** Fear, COVID-19, Older adults, FCV-19S, Bangladesh

## Abstract

**Objective:**

This study was aimed to assess the perceived fear of COVID-19 and its associated factors among older adults in Bangladesh.

**Methods:**

This cross-sectional study was conducted in October 2020 among 1032 older Bangladeshi adults aged ≥60 years. A semi-structured questionnaire was used to collect information on participants’ characteristics and COVID-19 related information. Perceived fear of COVID-19 was measured using the seven-item Fear of COVID-19 Scale (FCV-19S), where the cumulative score ranged from 7 to 35. Multiple linear regression was performed to identify factors associated with perceived fear of COVID-19.

**Results:**

The mean fear score was 19.4. Participants who were concerned about COVID-19 (*β*: 2.75, 95% CI: 1.71 to 3.78) and overwhelmed by COVID-19 (*β*: 3.31, 95% CI: 2.33 to 4.29) were significantly more likely to be fearful of COVID-19. Moreover, older adults who felt themselves isolated from others and whose close friends and family members were diagnosed with COVID-19 were more fearful. However, the participants who received COVID-19 related information from the health workers had a lower level of fear (*β*: -1.90, 95% CI: − 3.06 to − 0.73).

**Conclusions:**

The presence of overwhelming fear of COVID-19 among the older adults of Bangladesh underlines the psychological needs of these vulnerable groups. Health workers have a key role in addressing these needs and further research is needed to identify the effective strategies for them to use.

## Background

The emergence of COVID-19 has resulted an unprecedented disruption in both physical and mental health among the global citizens [1, 2]. Although many people have been impacted by the debilitating effects of COVID-19, certain groups, such as the older adults are more vulnerable than others and are at increased risk of severe morbidity and mortality due to the presence of comorbidities [3].

The recent evidence suggest that, the mortality rate from COVID-19 was 15% among older adults aged 80 years or above compared to only 0.2% among younger people aged less than 20 years and 74% of the total COVID-19 deaths occurred among those who were aged 65 years and above [4]. Serious emotional disturbances, insecurity, anxiety and depression are more common among old-aged people in association with social isolation, fear of uncertainty, and economic difficulties.

Previous outbreaks have been documented to be associated with a rise in mental illness among affected populations. For example, during the H_1_N_1_ influenza virus outbreak in 2009 in the United Kingdom (UK), there was a 10–30% increment in level of anxiety among the population [5]. Similarly, the SARS (severe acute respiratory syndrome) epidemic was also associated with increased depression, anxiety and psychiatric morbidities among the population [6]. The Ebola outbreak in Liberia, Guinea, and Sierra Leone during 2013–2016 impacted on the psychosocial wellbeing of people in affected areas [7]. It is, thus, not surprising that the COVID-19 pandemic has been associated with an increase in serious mental health issues such as anxiety, stress, insomnia, fear, anger and depressive symptoms in older adults [8].

Fear has been one of the most frequent emotions associated with the COVID-19 pandemic [9]. Uncertainty, worry, health anxiety, media exposure, personal health and the risk for loved ones are the predictors of fear for this disease [10]. Fear can affect older peoples’ mood or behavior and worsen their physical, social and cognitive functions [11, 12].

Similar to many other countries [13], the population of Bangladesh is rapidly ageing. Recent data suggest that there is more than 3.2 million people aged 60 years or above, comprising 7.7% of the total population in Bangladesh are aged 60 years and above which is projected to increase three-fold to 21.9% by 2050 [14].

Bangladeshi older adults are at increased risk of developing fear of COVID-19 for several reasons. Firstly, Bangladesh is one of the top countries in terms of the number of confirmed cases of COVID-19. As of 2nd January 2021, there were over 514 thousand reported COVID-19 confirmed cases and 7576 COVID-19 deaths in Bangladesh [15]. Secondly, older adults are at increased risk of severe illness and mortality due to COVID-19 than younger age groups [16]. Moreover, the prevalence of non-communicable chronic conditions i.e., hypertension, obesity, diabetes, cardiovascular disease, and chronic lung disease, are high among Bangladeshi older adults which increases their risk of severe health outcomes [17].

While it is presumed that COVID-19 might have created significant fear among the older adults, there is no data on perceived fear resulting from the ongoing COVID-19 pandemic in Bangladesh. Hence, the objective of this study was to explore the perceived fear of COVID-19 and associated factors among older Bangladeshi adults.

## Methods

### Study design and participants

Because of the contagious nature of the coronavirus and risk of spreading the virus through face-to-face contact, this cross-sectional study was carried out remotely through telephone interviews.

The study was conducted by Aureolin Research, Consultancy and Expertise Development (ARCED) Foundation in October 2020. We used our pre-established registry, developed through merging the contact information of households from different research projects accomplished by ARCED Foundation, which included households from all eight administrative divisions of Bangladesh, as a sampling frame.

We estimated sample size based on an unknown prevalence of fear (therefore considering 50% prevalence) with a 5% margin of error to be tolerated at the 95% level of confidence, 90% power of the test, and 95% response rate. On this basis, a total sample size of 1096 was required [18, 19]. However, a total of 1032 older adults aged 60 years and above agreed to participate in the study, resulting a response rate of approximately 94%.

We adopted a probability proportionate to number of older adults in each division to ensure representation from eight administrative divisions of Bangladesh [20]. The inclusion criterion was the minimum age of 60 years, and people with adverse mental conditions (clinically proved schizophrenia, bipolar mood disorder, dementia/cognitive impairment), a hearing disability, or inability to communicate were excluded.

### Measures

#### Outcome measure

COVID-19 related fear was the primary outcome, which was measured using the seven-item Fear of COVID-19 Scale (FCV-19S) developed and validated by Ahorsu et al. among the general Iranian population [21]. Participants’ agreement/disagreement with the seven items was assessed using a five-point Likert-scale (ranging from 1 = “strongly disagree,” 3= “neither agree nor disagree,” and 5 = “strongly agree”). Hence, the cumulative score ranged from 7 to 35, where the higher the scores, the greater the fear of COVID-19. Reliability or the internal consistency of the scale among the older adults was acceptable (Cronbach’s α = 0.89).

#### Explanatory variables

Explanatory variables considered in this study were age in year (categorized as 60–69, 70–79, and ≥ 80), sex (male/female), marital status (married/widowed), literacy (Illiterate/literate), family size (≤4/> 4), family income in Bangladeshi Taka (BDT) (< 5000, 5000-10,000, > 10,000), residence (urban/rural), current occupation (currently employed/unemployed and retired), living arrangements (living alone or with family), walking time to the nearest health center (< 30 min/≥30 min), memory or concentration problems (no problem/low memory or concentration), presence of pre-existing non-communicable chronic conditions (yes/no), concerned about COVID-19 (hardly, sometimes/often), overwhelmed by COVID-19 (hardly, sometimes/often), difficulty in getting food, medicine, and routine medical care during COVID-19 (no/yes), difficulty in earning during COVID-19 (no/yes), perceived isolation (hardly, sometimes/often), frequency of communication with friends and family during COVID-19 (less than previous/same as previous), receiving any financial support (government or non-government) during COVID-19, and source of COVID-19 related information (TV/Radio, health workers, and friends/family/neighbors).

Self-reported information on non-communicable chronic conditions, such as arthritis, hypertension, heart diseases, stroke, hypercholesterolemia, diabetes, chronic respiratory diseases, chronic kidney disease, and cancer were collected.

### Data collection tools and techniques

A pre-tested semi structured questionnaire in Bengali language was used to collect the information through telephone interview. The information was noted in SurveyCTO mobile app (https://www.surveycto.com/) by 10 research assistants, recruited based on previous experience of administering health survey in electronic platform. The research assistants were trained extensively for 3 days before the data collection by SKM, AMI and UNY through Zoom meeting.

The English version of the questionnaire was first translated to Bengali language and then back translated to English by two researchers to ensure its consistency. The questionnaire was then piloted among a small sample (*n* = 10) of older adults to refine the language of the questions in the final version. The questions used in the pilot stage did not require any corrections.

### Statistical analysis

The distribution of the variables was assessed using descriptive statistics. Independent t-tests and ANOVA evaluated the mean differences in the FCV-19S score by participants’ characteristics. A multiple linear regression model was performed to outline the factors associated with fear where backward elimination criteria with Akaike information criterion (AIC) was employed to select the final model. Adjusted beta-coefficient (*β*) and 95% confidence interval (95% CI) are reported for regression analysis. All analyses were performed using the statistical software package Stata (Version 14.0).

## Results

### Participants’ characteristics

As shown in Table 1, of the total 1032 participants, the majority were aged 60–69 years (77.8%), male (65.5%), married (81.4%), and had large families with more than 4 members (69.2%). Most of the participants were illiterate (58.3%), unemployed (59.4%), resided more than 30 min of walking distance from the nearest heath center (50.8%) and in rural areas (73.9%). Nearly half of the participants (46.1%) had a monthly family income of < 10,000 Bangladeshi Taka (BDT). Majority of the participants had pre-existing non-communicable chronic conditions (64%) and reported experiencing financial hardships during COVID-19 (62.7%) (Table 1).

**Table 1:** Participant characteristics and bivariate analysis (*N* = 1032)

Characteristics	n	%	COVID-19 fear score		
			Mean	SD	*P*
Total	1032	100.0	19.4	6.1	
Age (year, %)					
60–69	803	77.8	19.3	6.1	0.376
70–79	174	16.9	19.4	6.0	
> = 80	55	5.3	20.5	5.9	
Sex					
Male	676	65.5	18.9	6.1	0.001
Female	356	34.5	20.3	5.9	
Marital status					
Married	840	81.4	19.5	6.2	0.992
Widow/Widower	192	18.6	19.0	5.4	
Literacy					
Illiterate	602	58.3	19.4	5.9	0.962
literate	430	41.7	19.4	6.2	
Family size					
≤ 4	318	30.8	19.2	6.2	0.478
> 4	714	69.2	19.5	6.0	
Family monthly income in Bangladeshi Taka					
< 5000	145	14.1	20.4	6.4	0.014
5000-10,000	331	32.1	18.7	6.3	
> 10,000	556	53.9	19.6	5.8	
Residence					
Urban	269	26.1	19.4	5.6	0.932
Rural	763	73.9	19.4	6.2	
Current occupation					
Employed	419	40.6	19.3	6.4	0.511
Unemployed and retired	613	59.4	19.5	5.9	
Living arrangement					
Living with other family members	953	92.3	19.3	6.0	0.005
Living alone	79	7.7	21.3	6.1	
Walking distance to the nearest health centre					
< 30 min	508	49.2	19.1	6.1	0.107
≥ 30 min	524	50.8	19.7	6.0	
Having problem in memory or concentration					
No problem	782	75.8	19.0	6.0	< 0.001
Low memory or concentration	250	24.2	20.6	6.2	
Pre-existing non-communicable chronic conditions					
No	424	41.1	18.5	6.3	< 0.001
Yes	608	58.9	20.1	5.8	
Concerned about COVID-19					
Hardly	299	29.0	15.4	4.7	< 0.001
Sometimes/often	733	71.0	21.1	5.8	
Overwhelmed by COVID-19					
Hardly	370	36.4	15.8	5.2	< 0.001
Sometimes/often	647	63.6	21.4	5.5	
Difficulty getting food during COVID-19					
No	553	55.3	17.7	6.0	< 0.001
Yes	447	44.7	21.2	5.4	
Difficulty earning money during COVID-19					
No	340	37.4	16.9	5.9	< 0.001
Yes	570	62.6	20.8	5.7	
Difficulty getting medicine during COVID-19					
No	733	75.3	18.4	6.0	< 0.001
Yes	240	24.7	22.2	5.0	
Difficulty receiving routine medical care during COVID-19					
No	644	69.6	18.5	6.1	< 0.001
Yes	281	30.4	21.5	4.9	
Feeling of isolated					
Hardly	636	61.6	18.2	6.1	< 0.001
Sometimes/often	396	38.4	21.4	5.5	
Frequency of communication during the COVID-19					
Normal	598	58.0	18.6	5.7	0.080
Less than previous	434	42.1	20.5	6.4	
Close friend or family member diagnosed with COVID-19					
No	934	93.2	19.2	6.0	0.003
Yes	68	6.8	21.5	6.2	
Received any financial support during the COVID-19					
No	764	74.0	19.4	6.1	< 0.001
Yes	268	26.0	19.6	5.9	
Received COVID-19 related information from Radio/TV					
No	175	17.0	17.5	5.2	< 0.001
Yes	857	83.0	19.8	6.2	
Received COVID-19 related information from health worker					
No	936	90.7	19.3	6.3	< 0.001
Yes	96	9.3	20.2	2.2	
Received COVID-19 related information from friends/family/neighbours					
No	297	28.8	20.6	6.7	< 0.001
Yes	735	71.2	18.9	5.7	

### Fear of COVID-19 among the participants

**Fig. 1:**
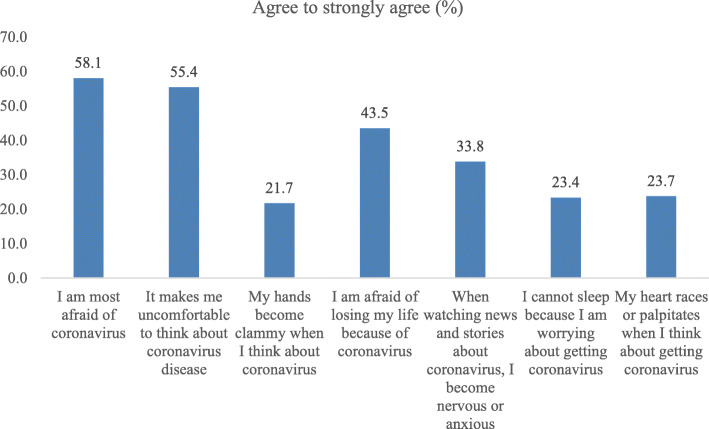
Participants agreement on the seven items of the COVID-19 fear scale

The mean score of the FCV-19S was 19.4 ± 6.1 among the participants. Mean differences in fear score of COVID-19 were noted by sex, family income, walking distance to the nearest health center, having problem in memory or concentration, non-communicable chronic conditions in Table 1. Participants’ reported agreement on the seven items of FCV-19S as shown in Fig. 1.

### Factors associated with the fear of COVID-19

Expressing concern about COVID-19, feeling overwhelmed by COVID-19 or experiencing difficulty in getting food or medicines, earning income and receiving routine medical care during the COVID-19 were all significantly associated with fear in the bivariate analysis. Feeling isolated, having a close friend or family member suffering from COVID-19, receiving any financial support during COVID-19, and receiving the information related to COVID-19 were also significantly associated with fear.

Socio-demographic, lifestyle and COVID-19 characteristics of the participants that are deemed to be associated with fear of COVID-19 (Table 1) were included in the full Regression model. The final model was selected based on the lowest AIC and is shown in Table 2. In the adjusted regression model, occupation, living arrangement, dependence on family for living, concern about COVID-19, overwhelmed by COVID-19, difficulty in earning during COVID-19, difficulty in getting medicine during COVID-19, feeling of isolated, close friend and family members suffering from COVID-19 and receiving COVID-19 related information for health workers were significantly associated with fear score (FCV-19S).

**Table 2:** Factors associated with fear among the participants (*N* = 1032)

Characteristics	β	95% CI	*P*
Sex			
Male	*Ref*		
Female	0.72	−0.17, 1.60	0.111
Current occupation			
Employed	*Ref*		
Unemployed	**0.88**	**0.02, 1.74**	**0.044**
Living arrangement			
Living with other family members	*Ref*		
Living alone	**2.01**	**0.67, 3.35**	**0.003**
Concern about COVID-19			
Hardly	*Ref*		
Sometimes/often	**2.75**	**1.71, 3.78**	**< 0.001**
Overwhelmed by COVID-19			
Hardly	*Ref*		
Sometimes/often	**3.31**	**2.33, 4.29**	**< 0.001**
Difficulty earning money during COVID-19			
No	*Ref*		
Yes	**1.04**	**0.21, 1.88**	**0.015**
Difficulty getting medicine during COVID-19			
No	*Ref*		
Yes	**2.16**	**1.29, 3.03**	**< 0.001**
Feeling of isolated			
Hardly	*Ref*		
Sometimes/often	**0.90**	**0.12, 1.68**	**0.024**
Close friend or family member diagnosed with COVID-19			
No	*Ref*		
Yes	**1.44**	**0.10, 2.79**	**0.036**
Received COVID-19 related information from health worker			
No	*Ref*		
Yes	**−1.90**	**−3.06, −0.73**	**< 0.001**

Fear scores were 0.88 units higher among participants who were currently unemployed (*β*: 0.88, 95% CI: 0.02 to 1.74), and 2.01 units higher who were living alone (*β*: 2.01, 95% CI: 0.67 to 3.35). Similarly, the fear scores were 2.75 units higher among those who were concerned about COVID-19 (*β*: 2.75, 95% CI: 1.71 to 3.78), 3.31 units higher who were overwhelmed by COVID-19 (*β*: 3.31, 95% CI: 2.33 to 4.29), 1.04 units higher who faced difficulty in earning during COVID-19 (*β*: 1.04, 95% CI: 0.21 to 1.88), and 2.16 units higher among those who had difficulty getting medicine during COVID-19 (*β*: 2.16, 95% CI: 1.29 to 3.03). The fear scores were 1.44 units higher among the participants whose close friend or family members were diagnosed with COVID-19 (*β*: 1.44, 95% CI: 0.10 to 2.79) and 0.90 units higher among those who felt isolated during COVID-19 (*β*: 0.90, 95% CI: 0.12 to 1.68). On the other hand, the fear score was 1.90 units lower among those who were receiving COVID-19 related information from the health workers (*β*: -1.90, 95% CI: − 3.06 to − 0.73).

## Discussion

The present study assessed the perceived fear of COVID-19 and its associates among Bangladeshi older adults. The findings of the study indicate that the COVID-19 pandemic created significant fear among the older population with a mean fear score of 19.4 on a seven-item fear scale (fear score ranged between 7 and 35). We also found a variation in participants’ agreement on the seven items of the COVID-19 fear scale, which might have resulted from different levels of perceived emotional responses to the phenomenology of the pandemic, such as cease and desist warnings, unending uncertainty, and concern over physiological symptoms [22, 23].

While there is scarcity of evidence on the level of COVID-19 related fear among older adults in Bangladesh, studies from other countries noted that COVID-19 has been associated with marked deterioration in the mental health of older adults due to anxiety about the lethality of COVID-19 [24, 25]. Our finding is also in line with some recent publications from Bangladesh that have reported COVID-19 related fear, psychosocial effects, and uncertainty among different groups of people [26–30].

Rapid spread of COVID-19 has seriously affected the mental health of the older people worldwide [24, 31]. Mental health problems have spiked among the older population mostly due to isolation and loneliness resulting from measures to control the spread of COVID-19 [32]. This suggests that we should pay more attention to the care and psychological support needs of the older population during this pandemic [33, 34].

The present study also highlighted the factors associates with COVID-19 related fear among the Bangladeshi older adults. We found that older adults who were currently unemployed or retired were significantly more likely to be fearful of COVID-19. This is probably because unemployed and retired person were less able to communicate with others as they did before the pandemic due to the lockdown and forced isolation during this pandemic. This may contribute to feelings of loneliness and isolation among them [35, 36]. We also found that the participants who had financial difficulty during this pandemic were more fearful of it. During the lockdown due to the COVID-19, many income earning members of the family lost their jobs in Bangladesh, particularly among the garments worker [37]. Also, crops and vegetables were not harvested in time or could not be transported to markets in time which caused food shortages and price increases [38]. Peoples earning are reducing day by day due to the disease outbreak on one side, on the other hand, the price of the foods are increasing, resulting in increasing fear among the participants.

Our study also found that older adults who were feeling isolated during this COVID-19 and those who were living alone had higher fear scores. Moreover, the participants who were concerned about the effect of the COVID-19 and were overwhelmed by it were more fearful than those who were indifferent to it. This is anticipated as fear of COVID-19 triggers psychological distress [39]. When the older adults know that they are more vulnerable to death and disability due to COVID-19 than their younger counterparts and that the treatments for COVID-19 are quite limited, they can become fearful of being infected with it [40, 41].

The present study also found that the older adults who face difficulties in getting medicine during this pandemic were more fearful. This is verified in a recently published evidence which documented that older adults from Low- and Middle- Income Countries faced various challenges including access to medicine and routine medical care amid this COVID-19 pandemic [42]. As in many other countries, during lockdown and isolation many people from Bangladesh faced problems accessing their daily essentials [43, 44]. For the older adults, medicines are essential especially as they often suffer from different comorbidities and in cases multiple morbidities [45]. The unavailability of required medicines can have serious impact on their mental health resulting fear.

Fear levels were high among the participants whose close friends and family members were diagnosed with COVID-19. This is expected especially among those older adults who suffered from pre-existing mental illness [46] and those whose friends or contacts were hospitalized with COVID-19.

Interestingly we found that the fear scores were significantly lower among the participants who received COVID-19 related information from health workers. This is probably because the health workers are trusted among older community members and provide information in a sympathetic manner [47].

### Strengths and limitations of the study

To the best of our knowledge, this is the first nationwide study covering all the eight administrative divisions of the country with high response rate and providing an insight into fear among Bangladeshi older adults amid this COVID-19 pandemic. However, our study has some limitations. As we prepared our sampling frame based on the available household-level information in our data repository; thus, selection bias is possible. Second, while we used FCV-19S as an instrument to measure fear among older adults, the efficacy of this instrument has not been tested and validated in Bangladeshi context. Finally, a mixed method study could have had better insights into the associated factors of fear.

## Conclusion

Mental health is one of the major issues among older adults which has been neglected in Bangladesh. Fear can be disabling during an unprecedented pandemic and may adversely affect their mental wellbeing. In the present study we also found that many older adults were fearful of COVID-19 and this was more likely in those with other social vulnerabilities such as low income, unemployment, or social isolation and who had difficulty accessing medications and health care. Health workers can be effective in providing information and psychosocial support to older adults as they are trusted members of the community.

The findings of the present research are also important for other countries where older adults face social and economic vulnerabilities, are subjected to social isolation during this pandemic and have to access to routine health services through a fragile health system. As the present research identified that socially and economically vulnerable older adults were more prone to be fearful of COVID-19, future work should focus on exploring strategies to minimize the socio-economic vulnerabilities among them. Providing statutory and informal economic support and strengthening community engagement to promote mental wellbeing among older adults can be of value in this regard.

## Data Availability

The data is available upon reasonable request from the corresponding author.
